# Postoperative hepatic arterial chemotherapy in high-risk patients as adjuvant treatment after resection of colorectal liver metastases - a randomized phase II/III trial – PACHA-01 (NCT02494973)

**DOI:** 10.1186/s12885-018-4697-7

**Published:** 2018-08-06

**Authors:** Diane Goéré, Jean-Pierre Pignon, Maximiliano Gelli, Dominique Elias, Léonor Benhaim, Frédéric Deschamps, Caroline Caramella, Valérie Boige, Michel Ducreux, Thierry de Baere, David Malka

**Affiliations:** 1 Department of Surgical Oncology - Gustave Roussy, 114 rue Edouard Vaillant, 94805 Villejuif Cedex, France; 2 Statistics and Epidemiology Unit - Gustave Roussy, Villejuif, France; 3 Centre for Research in Epidemiology and Population Health (team 2), INSERM U1018, Paris-Saclay University, Villejuif, France; 4 Department of Interventional Radiology - Gustave Roussy, Villejuif, France; 5 Department of Radiology - Gustave Roussy, Villejuif, France; 6 Department of Cancer Medicine - Gustave Roussy, Villejuif, France

**Keywords:** Colorectal cancer, Liver metastases, Liver resection, Adjuvant chemotherapy, Hepatic arterial infusion, Oxaliplatin, Randomized trial

## Abstract

**Background:**

After curative-intent surgery for colorectal liver metastases (CRLM), liver recurrence occurs in more than 60% of patients, despite the administration of perioperative or adjuvant chemotherapy. This risk is even higher after resection of more than three CRLM. As CRLM are mostly supplied by arterial blood flow, hepatic arterial infusion (HAI) of chemotherapeutic agents after resection of CRLM is an attractive approach. Oxaliplatin-based HAI chemotherapy, in association with systemic fluoropyrimidines, has been shown to be safe and highly active in patients with CRLM. In a retrospective series of 98 patients at high risk of hepatic recurrence (≥4 resected CRLM), adjuvant HAI oxaliplatin combined with systemic chemotherapy was feasible and significantly improved disease-free survival compared to adjuvant, ‘modern’ systemic chemotherapy alone.

**Methods/Design:**

This study is designed as a multicentre, randomized, phase II/III trial. The first step is a non-comparative randomized phase II trial (power, 95%; one-sided alpha risk, 10%). Patients will be randomly assigned in a 1:1 ratio to adjuvant systemic FOLFOX (control arm) or adjuvant HAI oxaliplatin plus systemic LV5FU2 (experimental arm). A total 114 patients will need to be included. The main objective of this trial is to evaluate the potential survival benefit of adjuvant HAI with oxaliplatin after resection of at least 4 CRLM (primary endpoint: 18-month hepatic recurrence-free survival rate). We also aim to assess the feasibility of delivering at least 4 cycles of HAI (or i.v.) oxaliplatin after surgical treatment of at least 4 CRLM, the toxicity (NCI-CTC v4.0) of adjuvant HAI plus systemic chemotherapy, including HAI catheter-related complications, compared to systemic chemotherapy alone, and the efficacy of adjuvant HAI on hepatic and extra-hepatic recurrence-free (survival and overall survival).

**Discussion:**

If 18-month hepatic recurrence-free survival is greater than 50% in the experimental arm, the study will be pursued in phase III, for which the primary endpoint will be 3-year recurrence-free survival rate. Patients randomized in the phase II will be included in the phase III, with an additional number of 106 patients.

**Trial registration:**

ClinicalTrials.gov, NCT02494973. Trial registration date: July 10, 2015.

## Background

### Adjuvant chemotherapy

Approximately 20% of patients with colorectal cancer (CRC) present with liver metastases (CRLM) at the time of diagnosis of the primary, and another 20% will develop CRLM during follow-up [1–4]. Surgical resection of CRLM is the only chance of cure – ant the best chance of long-term survival – and yields 5-year overall survival (OS) rates of 30 to 40% [5–7]. However, up to 60% of patients relapse following surgery, with recurrence confined to the liver in half of the cases [8, 9], even despite the administration of perioperative [10, 11] or adjuvant [12–15] chemotherapy.

A pooled analysis of two randomized trials (278 patients) comparing systemic 5-fluorouracil (5FU)-based adjuvant chemotherapy with no chemotherapy after complete resection of CRLM demonstrated a trend towards increased disease-free survival (DFS) for patients receiving chemotherapy (27.9 vs. 18.8 months; *p* = 0.095) [12]. In addition, in one of these trials, adjuvant chemotherapy was an independent favorable factor for DFS [13]. The EPOC trial studied the administration of FOLFOX chemotherapy versus no treatment before and after liver resection in patients with one to three colorectal liver metastases. In that trial, a significant disease-free survival benefit was observed in the treated group (per-protocol analysis), but not in the intention-to-treat analysis [10, 11].

### Hepatic arterial infusion of chemotherapeutic agents

Hepatic arterial infusion (HAI) has been developed to ensure greater local concentration of cytotoxic agents, since liver metastases derive most of their blood supply from the hepatic artery, while normal liver tissue is primarily perfused by the portal vein. Thus, HAI achieved significantly higher tumor response rates compared to systemic chemotherapy, as shown by several randomized studies in patients with unresectable CRLM [16–24].

Several different chemotherapeutic agents have been administered via HAI in the treatment of CRLM [25]. Fluorodeoxyuridine (FUDR) is mainly used for HAI because of its short half-life (< 10 min) and extensive first-pass extraction by the liver (94–99%) [26]. However, its biliary toxicity limits its administration. To improve the tolerance and efficacy of HAI with FUDR, the addition of steroid agents in the hepatic artery (in order to reduce biliary toxicity) and of cytotoxic drugs (e.g., irinotecan, oxaliplatin) systemically has been developed [27, 28].

The alternative is the use of HAI of more recent molecules. In this field, HAI with oxaliplatin had the most important development, mainly in France. We reported that HAI oxaliplatin accumulates in liver metastases with a tumor/normal parenchyma concentration ratio of 4.3 and a significant decrease in total platinum and ultrafiltrable platinum [29], suggesting potential benefit of the HAI route in terms of tolerance (e.g., peripheral neuropathy) and efficacy. HAI oxaliplatin also exhibited a liver extraction ratio of 0.47 [30]. After a Phase I trial conducted in Germany, we have shown in a multicenter Phase II trial that HAI oxaliplatin and systemic 5FU-folinic acid (LV5FU2 regimen) induced a response rate of 64% (95% CI: 44–81%) and a median overall survival of 27 months (survival at 1 and 2 years: 82 and 63%, respectively) in 28 patients with unresectable CRLM in the first line (*n* = 7) or second-line (*n* = 21) setting [31]. The combination was well tolerated with main toxicity consisting of grade 3 (*n* = 8) or 4 (*n* = 2) neutropenia and severe pain during the administration of oxaliplatin (*n* = 6).

More recently, we showed that the addition of HAI oxaliplatin to systemic chemotherapy succeeded in converting unresectable CRLM to resectable lesions in 24% of patients, with a complete pathological response rate of 19% in the patients who underwent surgery [32].

### Adjuvant HAI of chemotherapeutic agents

Since the majority of recurrences occurs in the liver, adjuvant HAI chemotherapy is an option after resection of CRLM. Several randomized studies have compared adjuvant HAI of chemotherapeutic agents to fluoropyrimidine-based systemic chemotherapy or to surgery alone, with conflicting and somewhat outdated results [33–41]. Specifically, DFS and/or hepatic DFS have been demonstrated to be superior with adjuvant HAI as opposed to systemic chemotherapy in five of the ten randomized studies performed to date. In these studies, FUDR was the main chemotherapeutic agent used for HAI. Kemeny et al. [36] reported the results from a single-institution study in which 156 patients were randomized to postoperative HAI with FUDR plus systemic 5-FU ± leucovorin vs systemic therapy alone. An increase in two-year survival rate for the combination therapy group was observed as compared with the control group (90% vs. 60%, *p* < 0.001). The liver relapse-free survival was also significantly increased in the combination therapy group. Furthermore, an updated analysis with a median follow-up of 10.3 years reported a significantly greater DFS rate (31.3 vs. 17.2 months, *p* = 0.02) and a trend toward improved OS (68.8 vs. 58.8 months, *p* = 0.10) in the combined therapy group compared to the control group [42]. In a more recent study, House et al. retrospectively analyzed 250 patients who underwent resection of CRLM between 2001 and 2005 and received either adjuvant HAI FUDR combined with systemic chemotherapy (FOLFOX or FOLFIRI regimen) or adjuvant systemic chemotherapy alone. The 5-year liver-recurrence free survival (RFS), overall RFS, and OS in the HAI group were 77, 48, and 75%, respectively versus 55, 25, and 55% in the systemic chemotherapy alone group (*p* < 0.01). The multivariate analysis also revealed adjuvant treatment with HAI plus systemic therapy as an independent factor for longer DFS (*p* < 0.01) [43]. Recently, the results of a phase II study (NCT00268463, NSABP–C-09) assessing the potential benefit of systemic oxaliplatin and capecitabine alternating with HAI of FUDR after resection of CRLM have been reported [44]. The primary end point was 2-year survival. Fifty-five of 76 eligible patients were able to initiate protocol-directed therapy and completed median of six cycles (range, one to six). Three postoperative or treatment-related deaths were reported. Overall, 88% of evaluable patients were alive at 2 years. With a median follow-up of 4.8 years, a total of 30 patients had disease recurrence, 11 involving the liver. Median disease-free survival (DFS) was 32.7 months. In conclusion, alternating HAI FUDR and systemic capecitabine and oxaliplatin met the prespecified end point of higher than 85% survival at 2 years and was clinically tolerable.

### Adjuvant HAI of chemotherapeutic agents in high-risk patients

The hepatic intra-arterial route requires more technicality than the venous route and should be reserved to patients at high risk of developing hepatic recurrence after resection of CRLM. In order to select these patients more at risk, based on the different prognostic scores [45–50], the factor common to all these scores and simple to establish, is the number of resected CRLM greater than or equal to 4.

We retrospectively analyzed 98 patients at high risk of hepatic recurrence (≥4 resected CRLM) treated postoperatively with either HAI oxaliplatin plus systemic 5-FU (*n* = 44) or ‘modern’ systemic chemotherapy (FOLFOX or FOLFIRI) (*n* = 54) [51]. Adjuvant oxaliplatin-based HAI chemotherapy was feasible, with more than four cycles of HAI administered in 84% of the patients (average number of HAI cycles, 8.0 ± 1.7). The 3-year hepatic DFS rate was significantly longer in the HAI group compared to the i.v group (49% vs. 21%, *p* = 0.0008), as was the 3-year DFS rate (33% vs. 5%, *p* < 0.0001). In multivariate analysis, adjuvant HAI chemotherapy and R0 resection margin status were the only independent prognostic factors for prolonged DFS. This study suggests that HAI oxaliplatin is feasible and significantly improves DFS in patients at high risk of hepatic recurrence after resection (or thermal ablation) of CRLM. The observed DFS benefit is sufficiently substantial to challenge the current standard of treatment and to warrant confirmation in a randomized trial targeting patients selected for their high risk of hepatic recurrence. To date, no randomized study has compared adjuvant HAI chemotherapy to ‘modern’ (i.e. oxaliplatin- or irinotecan-based) systemic chemotherapy while taking into account the risk of liver recurrence.

We believe that adjuvant HAI oxaliplatin after resection of high-risk CRLM is the ideal setting for implementing HAI techniques. Firstly, HAI oxaliplatin is administered in a 2-h infusion every 2 weeks – like via the i.v. route – compared to a 14-day infusion every 5 weeks with FUDR. Secondly, HAI oxaliplatin rarely causes chronic biliary toxicity, unlike FUDR.

Currently, no adjuvant study with HAI in the adjuvant setting is ongoing. HAI oxaliplatin plus systemic LV5FU2 has shown activity as first-line palliative treatment of CRLM. This raises the question whether this treatment could be of value as an adjuvant treatment after CRLM resection.

## Methods/ design

This study is designed as a multicenter, randomized phase II/III trial. The first step is a non-comparative randomized phase II trial. Its main objective study is to assess the efficacy of HAI oxaliplatin plus systemic fluoropyrimidine (LV5FU2 regimen) after curative-intent surgery (resection and/or thermal ablation) of at least 4 CRLM. Depending on the results, this randomized phase II study will be expanded into a phase III study to demonstrate the superiority of adjuvant HAI chemotherapy compared to systemic chemotherapy. The phase III will include the patients of the phase II and an additional group of patients to reach the total sample size needed for the phase III. If confirmed, this will have a clinically relevant impact on patient survival and an impact on public health because of the frequency of CRLM.

### Study objectives and endpoints

#### Primary objective

For the phase II, the primary objective is to assess the efficacy of HAI oxaliplatin plus systemic fluoropyrimidine (LV5FU2 regimen) after curative-intent surgery on 18-month hepatic recurrence-free survival (RFS) in patients at high risk of hepatic recurrence, meaning after resection and/or thermal ablation of at least 4 CRLM.

For the phase III, the primary objective is to demonstrate the superiority of adjuvant HAI oxaliplatin plus LV5FU2 compared to systemic oxaliplatin plus LV5FU2 (FOLFOX) on RFS in the same population.

#### Secondary objectives

The secondary objectives for phase II and III are based on the assessment of:

- the feasibility of delivering at least 4 cycles of HAI (or IV) oxaliplatin after surgical treatment of at least 4 CRLM.
- the toxicity of adjuvant HAI or of systemic chemotherapy after surgical treatment of at least 4 CRLM, including HAI catheter-related complications.
- the efficacy of adjuvant HAI plus LV5FU2 on RFS and OS and on the pattern of failures.

Hepatic RFS will be measured from the date of randomization to the date of hepatic recurrence, the date of other recurrences in the absence of hepatic recurrence as first event, the date of death whatever its cause in the absence of recurrence, or the date of last follow-up if the patient is alive. Non-hepatic recurrence as first event will be censored. RFS and OS will be measured from the date of randomization. For OS, the delay to the date of death, regardless of the cause, or to the date of last follow-up for patient alive will considered. For RFS, the delay to the date of recurrence, or death, regardless of the cause, or to the date of last follow-up for patient alive without recurrence will considered.

### Study population

This study will include patients after R0/R1 resection and/or thermal ablation of at least 4 CRLM (histological confirmation for at least one metastasis) without extrahepatic disease (except ≤3 lung nodules < 10 mm on chest CT scan deemed amenable to curative-intent resection/ablation).

Patients to be included in the study must fulfill the following inclusion criteria: histologically confirmed stage IV CRC, curative-intent R0/R1 resection (or thermal ablation) of at least 4 CRLM, preoperative oxaliplatin- and/or irinotecan-based chemotherapy +/− non-experimental biological therapy (e.g., anti-EGFR or antiangiogenic agent), confirmed radiological tumor control before surgery (i.e., objective response or stable disease according to RECIST1.1), age > 18 years, good health status (WHO performance status 0–1), adequate hematological function (absolute neutrophil count > 1.5 × 10^9^/l, platelets > 100 × 10^9^/l, hemoglobin > 9 g/dl), adequate liver function (serum bilirubin < 1.5 x ULN; alkaline phosphatase and transaminases < 5 x ULN), serum creatinine < 1.5 x ULN, informed consent signed by the patient or his/her legal representative, no pregnancy or breast feeding, adequate contraception in fertile patients, adequate private or national insurance coverage. Exclusion criteria include: extrahepatic tumor disease (except ≤3 lung nodules ≤10 mm on chest CT scan deemed amenable to curative-intent resection/ablation), symptomatic primary tumor requiring urgent surgery, contraindication to fluoropyrimidines or oxaliplatin, disease progression under oxaliplatin (including early hepatic relapse (less than 6 months) after end of adjuvant FOLFOX following primary tumor resection), history of any HAI treatment (chemotherapy, radioembolisation…), peripheral neuropathy> grade 1, history of cancer within 5 years prior to entry into the trial other than adequately treated basal-cell skin cancer or in situ carcinoma of the cervix, concomitant medications/comorbidities that may prevent the patient from receiving study treatments, patient already included in another clinical trial with an experimental molecule, patients unable to undergo medical monitoring test for geographical, social or psychological reasons.

An asymptomatic primary tumor is not a non-inclusion criterion if its resection is planned (reverse strategy allowed).

### Treatment schedule

Randomization will be either performed during surgery, or within 6 weeks after surgery using web-based procedure (TenAlea®). Adjuvant chemotherapy must begin within 8 weeks after surgery. Patients will be randomly assigned using minimization procedure in a 1:1 ratio to:

- Arm A (control arm): adjuvant systemic (i.v.) chemotherapy (FOLFOX) administered every 14 days:
  - Oxaliplatin 85 mg/m^2^ in 2 h IV day (D)1,
  - Folinic acid 400 mg/m^2^ in 2 h IV (concomitantly to oxaliplatin) D1, followed by
  - 5FU bolus 400 mg/m^2^ in 5–10 min IV D1 followed by
  - 5FU 2400 mg / m^2^ IV in 46 h
- Arm B (experimental arm): adjuvant HAI chemotherapy plus systemic chemotherapy (LV5FU2) administered every 14 days:
  - Oxaliplatin 85 mg/m^2^ in 2 h HAI D1,
  - leucovorin 400 mg/m^2^ in 2 h IV (concomitantly to oxaliplatin) D1, followed by
  - 5FU bolus 400 mg/m^2^ in 5–10 min IV D1 followed by
  - 5FU 2400 mg / m^2^ IV in 46 h

Randomization will be stratified according to the following factors:

- Tumor response to preoperative chemotherapy (objective response vs. stable disease)
- Number of resected CRLM (4–8 vs. > 8)
- Center

Patients will receive adjuvant chemotherapy (HAI or systemic) for a maximal duration of 6 months and at least 3 months for the postoperative period. The minimal duration of chemotherapy (pre- and postoperative period) will be of 6 months. Before starting adjuvant chemotherapy, CT scan of the abdomen, pelvis and chest and serum tumor markers will be done (within 4 weeks before starting adjuvant chemotherapy). In both arms, continuation of targeted therapy (if any) used in the preoperative treatment will be allowed.

The HAI catheter will be placed before initiating treatment, either surgically or percutaneously by interventional radiology and bound to an implantable port. Angiographic and/or scintigraphic verification of the HAI catheter functionality will be done within 28 days before the start of treatment. HAI chemotherapy should be performed by physicians and nurses familiar with this technique.

### Assessments and follow-up

Follow-up will include every 3 months for the first 3 years following surgical procedure (months 3 to 36) and twice a year for at least 2 years (months 42 to 60), and then once a year for 3 years. For each visit, the assessments described in Table 1 should be performed. To study long-term effect on OS, patients will be followed for at least 3 years. A clinical study report will be issued for the 3-year RFS study (see statistical analysis for timing).

**Table 1:** Plan of the study

VISITS	PRE-RANDOMIZATION WORKUP (maximum 1 month before)		Randomization	Follow-up during treatment					Follow-up		
				Every 2 weeks, during at least 3 months					The first three years : every 3 months	From 4 th to 5 th year : every 6 months	From the 6 th year to the8th : Every year
Visits N° Dates (days (D), months (M))	D-28 to D0	D-7 to D-1	D0	V1	V2	V3	V4	Vn	M 3, M6, M9, M12,....	M43, M49, M55, M61	
Informed consent signed	X										
Inclusion/Exclusion criteria		X									
Surgery			X ^c^								
TREATMENT											
- Oxaliplatine IAH or IV				X	X	X	X	X			
- LV5FU2 IV				X	X	X	X	X			
CLINICAL EXAMINATION											
- Weight, BMI, OMS statut		X		X	X	X	X	X	X	X	X
- Treatment toxicity				X	X	X	X	X	X		
EXAMS											
- Thoraco-Abdomino-pelvic CT scan^d^	X								X	X	X
- Electrocardiogram (ECG)		X									
- Control of the arterial catheter^a^				Every 8 weeks or more frequently if deemed necessary by the physicians							
LABORATORY EXAMS^b^											
- NFS-platelets	X			X	X	X	X				
- PT, INR	X										
- Ionogram, urea, creatinin level, liver biology	X	X		X	X	X	X				
- ACE, CA 19-9	X								X	X	X
- Pregnancy test	X										

^a^radiological ou angioscintigraphy

^b^liver biology: transaminases, alcalin phophatase, gamma glutamyl transferase, bilirubin Within 4 weeks before starting adjuvant chemo

^c^Patients are randomized peroperatively or within 6 weeks after surgery

^d^completed by MRI and/or Petscan according to the physician

### Statistical considerations

#### Required number of patients

The phase II is based on a two-step optimum Simon design [52] for the experimental arm, with the same number of patient in the control arm. The control arm allows checking that patients included are comparable to those included in previous studies that led to build study hypotheses, and to expand this study in a Phase III study by using a phase II-III design, depending on the study results.

The hypotheses are the following:

- 18-months hepatic RFS rate with the control treatment of 30%
- 18-months hepatic RFS rate with the experimental treatment of 50%
- A minimum follow-up time of 18 months for patient alive without recurrence.

With this hypothesis and to have a 95% power (beta risk = 5%) and a one-sided alpha risk of 10%, a total of 108 patients will have to be randomized. Since 5% of patients will be non-evaluable, a total of 114 patients will be included. The rate of non-evaluable patient will be monitored and if necessary an increase in sample size will be proposed. After the inclusion of the first 30 evaluable patients, an analysis on safety and feasibility after 6-month follow-up will be performed and reported to the independent data monitoring committee (IDMC).

An interim efficacy analysis in both arms will be performed after the inclusion of the first 30 evaluable patients with a minimum of 18-month follow-up in the experimental arm as planned by the Simon design. This analysis will be reported to the IDMC. Among the first 30 evaluable patients in the experimental arm,

- If 7 on the experimental arm or fewer patients were free of hepatic recurrence at 18 months, the Phase II will be stopped because of poor efficacy and the trial will be stopped.
- If 8 or more patients on the experimental arm were free of hepatic recurrence at 18 months, the Phase II will continue and the Phase III (or its activation) will be continued.

After evaluation of 18-month RFS in 54 evaluable patients,

- if 20 or less patients out of the 54 of the experimental arm were free of hepatic recurrence, the trial will stop because of poor efficacy.
- Otherwise, the conclusion will be that the 18-month RFS is good enough to continue or open to accrual the Phase III study.

For the Phase III, the primary endpoint will be 3-year recurrence-free survival (RFS) rate. Patients randomized in the Phase II will be included in the Phase III. The hypotheses are the following:

- 3-year RFS rate with the control treatment of 15%
- 3-year RFS rate with the experimental treatment of 30%, (HR 0.63)

With this hypothesis (α- risk, 5% (two-tailed); β- risk, 20%), the corresponding number of patients is 204 patients (152 events). With an increase of 7–8% (ineligible patients etc.…), the total number of patient is: 220 (164 events), i.e. 110 patients/arm, including 106 additional patients in the Phase III.

#### Statistical analysis plan

The phase II analysis will be conducted on all the patients registered and randomized, in “intention to treat”. A second analysis will be conducted on the “treated” population, determined according to the treatment actually administered (per protocol analysis). The phase III analysis will be performed according to intent-to-treat principle, i.e. on all patients randomized. A minimum follow-up of 18 months for the last enrolled patient will be required.

The results for the primary and secondary endpoints will be presented by arm with a confidence interval at 95% (Rothman for survival data). Compliance data will be reported by number of cycles. The total dose of 5-FU (overall and according to its modalities: (bolus, continuous perfusion) by square meter, as well as the total dose of oxaliplatin (overall and according to its modalities: IA and IV) and the type and dose of target therapy if any will be described. The corresponding dose-intensity will be computed. Information on the treatment of recurrence will be also collected. Safety data will be reported according to their frequency and by system organ class. Analysis per patient (maximum grade) and per cycle will be reported. The proportion of patient with at least one grade 3 or more toxicity will be computed. For overall survival, survival rates at 12 and 24 months and median will be calculated. For hepatic and overall RFS, rates at 12 and 18 months and median will be calculated. Pattern of recurrences will be also described. As the trial is constructed as a phase II, no statistical test will be made.

The analysis on the 3-year RFS, the primary endpoint for the phase III, will be performed once the number requested of events will be reached and a median follow-up of at least 3 years observed. A long-term follow-up analysis with a minimum follow-up of 3 years and a median follow-up of at least 5 years will be also performed. Main endpoint and secondary efficacy endpoint will be compared by logrank test and reported with a hazard ratio and its 95% confidence interval. Data on compliance and safety will be compared by Wilcoxon or Chi^2^ test as appropriate.

### Toxicity monitoring

Intensity of events will be estimated according to the NCI-CTCAE classification, version 4.0 (toxicity score grade 1 to 5). Catheter-related complications will be specifically evaluated.

## Discussion

This study is important as it provides proof of concept for the potential role of adjuvant chemotherapy with HAI oxaliplatin in patients who have undergone curative resection of liver metastatic disease. All the available data in the literature and the observed DFS benefit in the previous retrospective study [51] suggest that it could be interesting to evaluate adjuvant hepatic arterial infusion with oxaliplatin plus systemic 5-FU after resection of at least 4 CRLM, in order to decrease the rate of hepatic recurrence.

Regarding the feasibility, the main observed side effects related to the intra-arterial administration of oxaliplatin and which can limit the total dose of treatment are: 1) cumulative peripheral neuropathy, which can lead to stop HAI treatment while continuing the systemic treatment, as in the case of adjuvant IV chemotherapy; 2) extra-hepatic diffusion of chemotherapy (most often manageable by percutaneous embolization of hepatic collateral vessels) that may cause gastroduodenal ulcerations; 3) abdominal pain during intra-arterial infusion, which is a specific complication of oxaliplatin. However, feasibility and toxicity related to HAI chemotherapy could be due to a lack of experience in this route of chemotherapy. Because of this and for the purpose of training teams to participate in this trial, biannual educational seminars on HAI are organized in Gustave Roussy since 4 years, bringing together oncologists, radiologists, surgeons and nurses.

Increasing local delivery of chemotherapy to the liver via the HAI route after resection of CRLM in patients at high risk of hepatic recurrence appears to be an attractive and promising option. To date, there is no controlled phase 3 trial comparing HAI to the “modern” (i.e. oxaliplatin- or irinotecan-based) systemic chemotherapy, and we have enough arguments in the literature to evaluate the potential benefit of adjuvant HAI in a randomized trial focused on patients at high risk of hepatic recurrence.

## Abbreviations

CRC: Colorectal cancer
CRLM: Colorectal liver metastases
D: Day
DFS: Disease-free survival
FUDR: Fluorodeoxyuridine
HAI: Hepatic arterial infusion
IV: Intravenous
OS: Overall survival
RFS: Recurrence-free survival
